# Immediate Loading of Single Implants in the Anterior Maxilla: A 1-Year Prospective Clinical Study on 34 Patients

**DOI:** 10.1155/2017/8346496

**Published:** 2017-05-22

**Authors:** Miguel Stanley, Filipa Calheiros Braga, Beatriz Mota Jordao

**Affiliations:** White Clinic, Rua Dr. António Loureiro Borges, Edifício 5, 1 Andar 131, 1495 Algés, Portugal

## Abstract

**Purpose:**

To present the outcomes of immediately loaded single implants placed in the anterior maxilla.

**Methods:**

Over a 2-year period, all patients referred to a private clinic were considered for enrolment in this study. Inclusion criteria were single-tooth placement in postextraction sockets or healed sites of the anterior maxilla. All implants were immediately loaded and followed for a period of 1 year after the placement of definitive crowns. The outcome measures were implant stability, survival, and success.

**Results:**

34 patients were selected and 43 tapered implants with a knife-edge thread design and a nanostructured, calcium-incorporated surface (Anyridge®, Megagen, Gyeongsang, Korea) were installed. Two implants were not sufficiently stable at placement (ISQ < 60) and were considered failed for immediate loading; 41 implants had an ISQ ≥ 60 at placement and were immediately loaded. One year after the placement of definitive crowns, no implant failures were reported, for a survival rate of 100%. No biological complications were found, but 2 implants had their prosthetic abutments loosened: the implant success rate was 95.2%.

**Conclusions:**

In the present study on the immediate loading of single implants in the anterior maxilla, positive outcomes were reported, with high survival (100%) and success (95.2%) rates* (the present study has been registered in the ISRCTN registry, a publicly available trial register recognized by WHO and ICMJE, with number *ISRCTN12935478).

## 1. Introduction

Dental implants are a viable solution for the restoration of single-tooth gaps, with high survival and success rates in the short [1] and long term [2]. Nowadays, the placement of an implant-supported single crown allows the rapid and predictable restoration of function (mastication) and aesthetics [3, 4].

A good biological integration is an essential prerequisite for the success of a fixed implant-supported restoration [5]. In fact, a dental implant has to effectively integrate into the bone, in order to functionally support the prosthetic restoration [5]; at the same time, of fundamental importance is the integration with the soft tissues, which is a guarantee of the maintenance of osseointegration over time, and it is an essential condition for the aesthetic success of the rehabilitation [4–6].

In recent years, the aesthetic requirements of the patients have become increasingly important and difficult to satisfy [4–6]; furthermore, patients require a treatment that should be fast, minimally invasive, and of low cost [5].

In order to meet the modern needs of patients, new surgical and prosthetic protocols have been proposed and are gaining acceptance, which reduce the number of operating sessions (and with them the stress and costs for the patient): among them, there are the placement of implants in fresh extraction sockets [4, 7, 8] and the immediate prosthetic loading [9].

The placement of implants in fresh extraction sockets, that is, immediately after the extraction of the nonrestorable, compromised teeth, can reduce the number of surgical sessions (from two to one) with a reduction in the patients' stress and costs [4, 7, 8]. This strategy is compatible with the insertion of implants with a flapless technique (i.e., without having to raise a full-thickness, mucoperiosteal flap) and is therefore minimally invasive: this represents a further advantage of the method [8, 10]. Finally, some researchers believe that the insertion of an implant into a fresh extraction socket may facilitate the correct three-dimensional (3D) positioning of the fixture, with benefits for the emergence profile [4, 7, 8].

Although all these benefits of immediate implant placement have been recognized, this surgical technique does not allow (in contrast to what had been assumed in the past) a reduction or counteracting of the physiological resorption that occurs in the alveolar bone after tooth extraction [11, 12], and that particularly affects the delicate buccal bone plate in the anterior maxilla [12]. In addition, the placement and primary stabilization of an implant in a fresh postextraction socket (which is generally larger) can be technically difficult and can be a challenge for the surgeon [7, 8, 13]. If the implant is placed too buccally, the final aesthetic outcome can be compromised [8, 13]; if the implant is placed too palatally (lingually), this situation may not be compatible with adequate prosthetic emergence profile [8, 13].

The immediate prosthetic loading is a viable strategy to reduce the time of treatment: the placement of a temporary restoration immediately after the insertion of the fixture (within 48–72 hours after surgery) is certainly an aesthetic and functional benefit to the patient, who can avoid wearing uncomfortable removable dentures during the healing period [9]. Finally, the placement of an immediate provisional restoration involves benefits with respect to gingival tissues, which can be modeled around it immediately [4, 6, 14].

However, there is a risk to be calculated in immediate loading of dental implants, especially when they support single crowns [15, 16]: in order to obtain a valid osseointegration, it is indeed necessary that the forces applied on the system in the early healing period are controlled, and they do not generate micromotions [17]. The presence of micromovements at the interface between bone and implant can, in fact, affect bone healing and osseointegration, leading to a mobilization and failure of the implant [17, 18].

In order to meet the new challenges of modern implantology, the manufacturers now offer implant systems with specific designs (macrotopographies) and surfaces (micro/nanotopographies) that can help to maximize the primary stabilization in difficult contexts (such as the placement in postextraction sockets) and at the same time speed up and enhance osseointegration, in order to anticipate the prosthetic loading without risk [19–22].

The aim of this prospective clinical study is therefore to present the clinical outcomes of single implants with a knife-edge thread design and a nanostructured calcium-incorporated surface, when placed in postextraction sockets and healed sites of the anterior maxilla and subjected to immediate loading.

## 2. Materials and Methods

### 2.1. Study Sample

The patients were enrolled in this prospective study and treated with the insertion of dental implants in the course of two years (2013-2014) in one private dental center (White Clinic®, Lisbon, Portugal). Inclusion criteria were as follows: (1) patients with one to four single-tooth gaps or patients in need of replacement of one to four severely compromised, nonrestorable teeth in the anterior areas of the maxilla (incisors, canines, and first and second premolars); (2) good state of systemic health; (3) good oral hygiene; (4) age > 18 years; (5) dentition in the opposite arch; (6) willingness to participate in the follow-up study, attending all annual periodic examinations/controls. The general exclusion criteria included the presence of medical conditions that contraindicated surgery, such as (1) uncontrolled or not properly treated diabetes with high blood sugar levels, (2) the presence of immunosuppression, (3) history of head and neck cancer with radio- and chemotherapy, (4) the presence of blood diseases, (5) the presence of psychological or psychiatric diseases, (6) patients in treatment with anticoagulants, and (7) patients in treatment with oral/intravenous aminobisphosphonates. The local exclusion criteria were as follows: (1) the absence of enough bone to place an implant of at least 10.0 mm in length and 3.5 mm in diameter; (2) the need of major regenerative bone techniques (such as onlay/inlay bone grafting) before implant insertion (minor procedures including guided bone regeneration with granulate and membranes or buccal grafting and interproximal procedures were not exclusion criteria); (3) the presence of oral diseases (vesiculobullous diseases, ulcerative diseases, white or red lesions, diseases of the salivary glands, the connective tissue or lymphoid lesions, cystic lesions, and benign or malignant tumors of the oral cavity); (4) the lack of occlusal contacts in the antagonist arch. History of periodontal disease, the habit of cigarette smoking, and the presence of parafunctions were not exclusion criteria for this study; however, patients were advised that these conditions could represent a risk factor for implant therapy [23]. All patients were informed in detail about the nature of this study and signed informed consent for implant therapy. The present study was carried out in full compliance with the criteria established by the Declaration of Helsinki on clinical trials involving human subjects (2008).

### 2.2. Preoperative Evaluation

**Figure 1:**
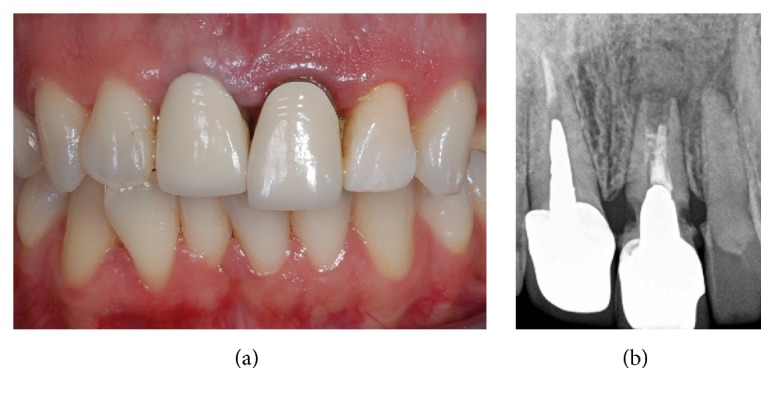
Preoperative situation. The patient complains because the left central incisor, which was restored with a single crown several years before, appears extruded and presents a high mobility (a). The periapical radiograph shows a severe resorption (b): the tooth is nonrestorable and needs to be extracted.

**Figure 2:**
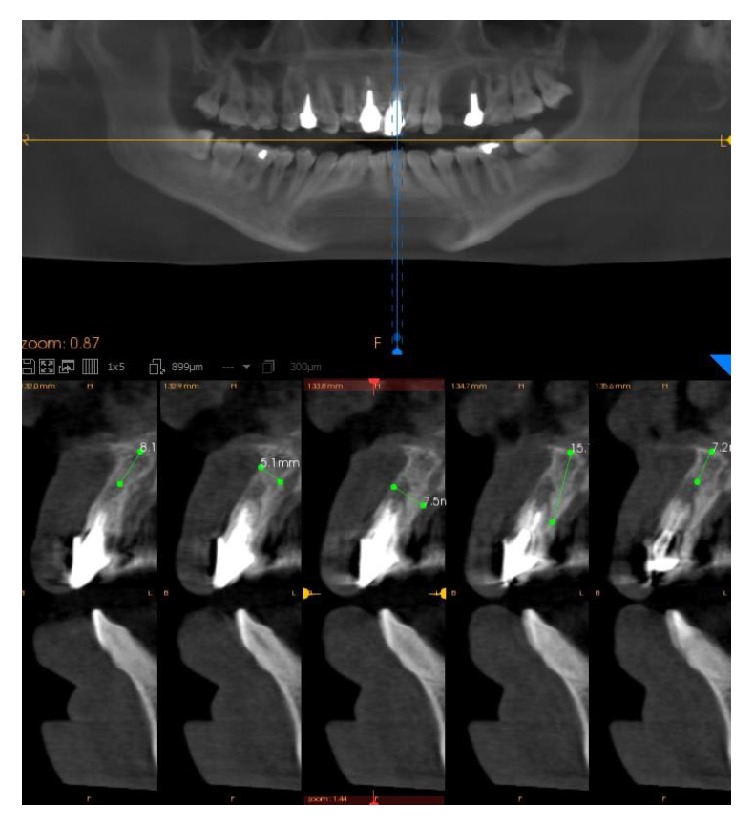
Cone beam computed tomography (CBCT). The cone beam computed tomography (CBCT) examination confirms the presence of the tooth resorption. A careful 3D analysis of the height and width of the alveolar ridge is performed, in order to better plan the implant placement.

The preoperative evaluation included a careful clinical and radiographic analysis (Figures 1 and 2). In particular, all patients underwent two-dimensional radiographic evaluation (intraoral periapical radiographs or panoramic radiograph) for a first assessment of the surgical site; when requested, this assessment was supplemented by a three-dimensional (3D) evaluation of bone anatomy by means of a low-dose cone beam computed tomography (CBCT) (CS9300®, Carestream Health, Rochester, USA). The DICOM files resulting from the CBCT were then loaded into visualization software, in order to evaluate in detail the height and thickness of the bone crest. The surgical planning then proceeded through a simulation of implant placement: this was helpful for deciding the length and diameter of the different fixtures and to better study location, depth, and inclination of the same fixtures. Radiographic evaluation was completed by taking two alginate impressions and pouring of plaster models, on which the dental technician made a diagnostic to wax-up, in order to better understand the patient's prosthetic needs.

### 2.3. The Implants

The tapered implants used here (Anyridge, Megagen, Gyeongsang, Korea) had a knife-edge thread design and a nanostructured, calcium-incorporated surface. The nanostructured surface of these implants (Xpeed®) was the result of a conventional sandblasting procedure (resorbable blast media treatment) and the subsequent incorporation of calcium ions by means of a hydrothermal method [24]. The implants had a 5 mm deep conical connection (10°) combined with an internal hexagon [6, 15, 20] and were available in different lengths (7.0, 8.5, 10.0, 11.5, 13.0, and 15.0 mm) and diameters (3.5, 4.0, 4.5, 5.0, 5.5, and 6.0 mm).

### 2.4. Surgical and Prosthetic Procedures

**Figure 3:**
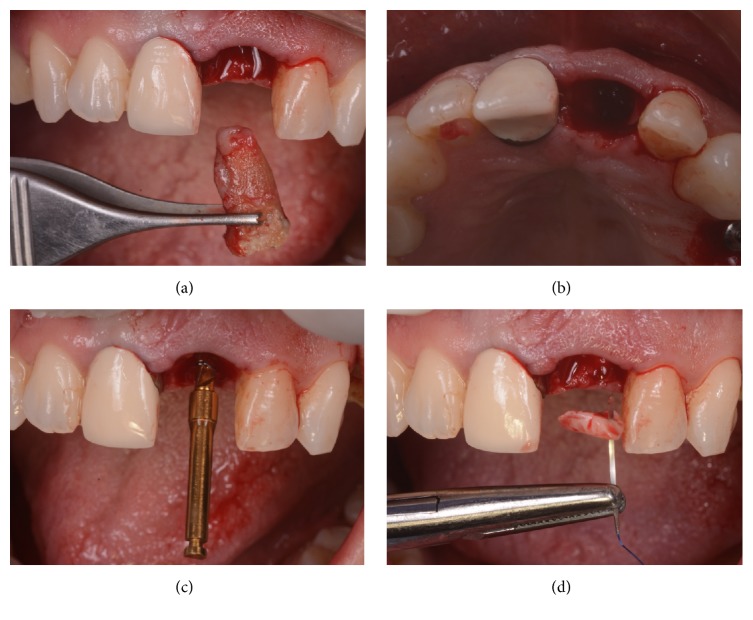
Surgery. The nonrestorable tooth is extracted (a) and the socket is carefully debrided (b), in order to remove all infected tissue; then, the implant site is prepared with sequential drills, exceeding the alveolar apex 3-4 mm (c) and before to place the implant, a connective tissue graft is harvested from the palate, in order to thicken the soft tissues in the delicate buccal area (d).

**Figure 4:**
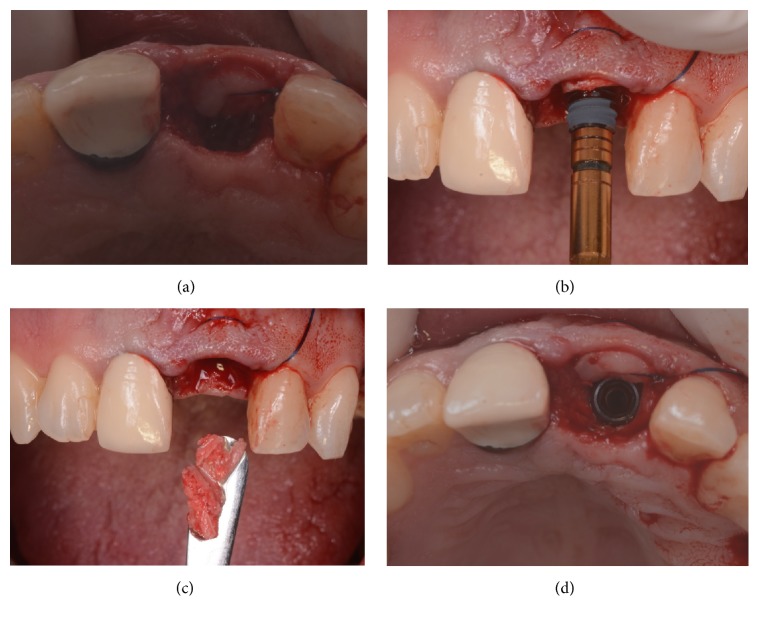
Surgery. The connective tissue graft is secured in position, within the envelope flap (a); then the implant (Anyridge, Megagen) is inserted slightly subcrestal and in palatal position (b); the autogenous bone chips collected during the preparation of the implant site are then placed in the alveolus (c), in order to fill the gaps between the socket and the implant (d).

**Figure 5:**
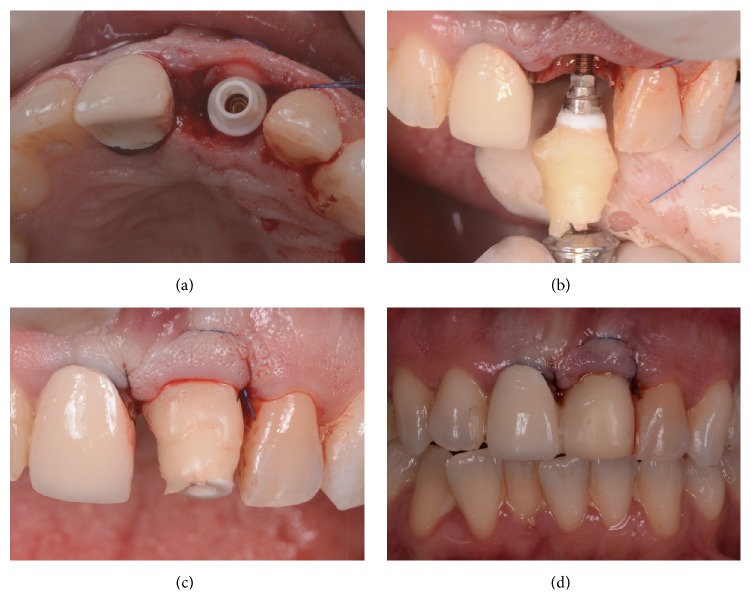
Immediate provisionalization. A provisional abutment is screwed on the implant (a) and the individual emergence profile is obtained with composite resin (b) in order the correct pressure that is exerted on the soft tissues (c); in this case, the immediate temporary restoration is then splinted to the adjacent teeth, for a short period after the surgery, in order to reduce the effects of prosthetic loading on the immediately inserted implant (d).

**Figure 6:**
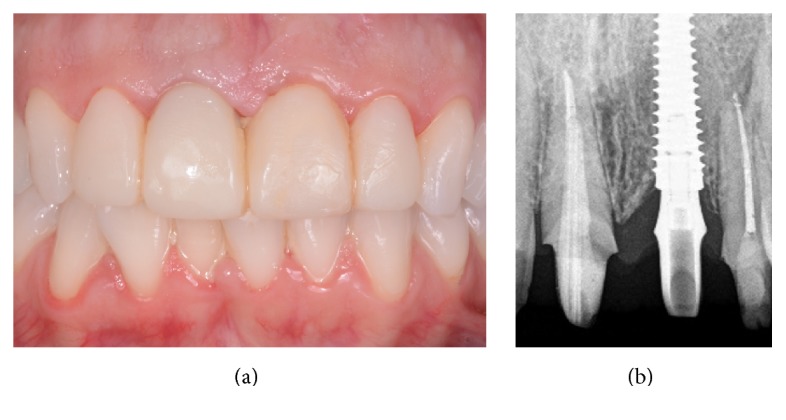
The provisional restoration after 2 months. The soft tissues outline has been modeled by the temporary and the level and curvature of the facial mucosa look better, even if oral hygiene should be improved. (a) Clinical view; (b) radiographic control.

**Figure 7:**
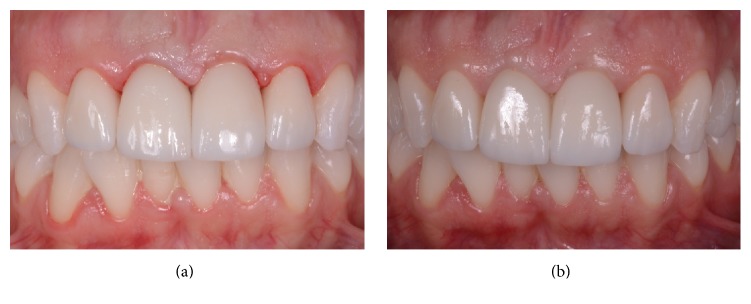
The definitive ceramic restoration in position. (a) The definitive ceramic crown is delivered to the patient, along with the other planned definitive, tooth-supported restorations. (b) The aesthetic result 4 months after implant placement.

**Figure 8:**
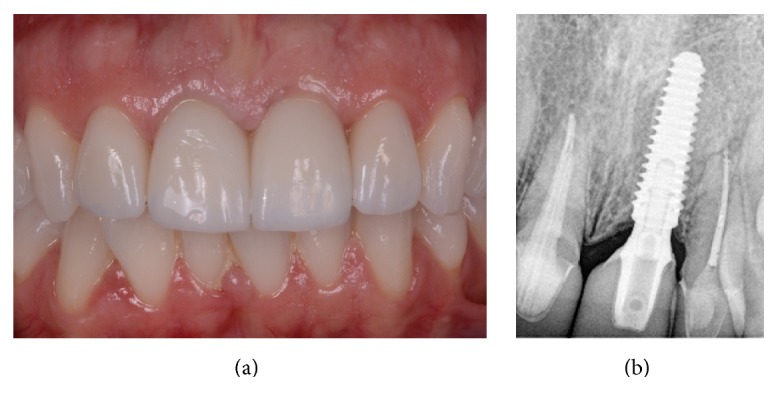
The definitive ceramic crown 1 year after the delivery. (a) An aesthetically pleasing result has been maintained clinically, and the patient is fully satisfied; (b) the radiographic control confirms the stability of the hard tissues around the implant.

All surgeries were performed under local anesthesia, using articaine with adrenaline (1 : 100,000) by the same experienced clinician (M.S.). In the case of single-tooth gaps in healed ridges, a midcrestal incision was performed connected with two lateral releasing incisions; a full-thickness flap was raised; then the surgeon prepared the implant sites using drills of increasing diameter, strictly following the manufacturer's recommendations. In the case of nonrestorable teeth that had to be extracted, the extraction was performed gently with the purpose of avoiding any damage to the buccal bone wall; the socket was carefully cleaned and the integrity of the socket walls was verified. Then, the surgeon prepared the implant site, without rising any flap apically and pushing 3 to 4 mm to the peak of the postextraction socket. In cases with high aesthetic demands (such as the central and lateral incisors) care was taken to prepare the implant site palatally, in order to avoid any contact with the delicate and thin buccal wall. In postextraction cases, after the insertion of the implants, the gaps between the fixture and the alveolus walls were filled with autogenous bone chips, recovered during the preparation of the surgical site (Figures 3 and 4); the autogenous bone could be mixed with highly porous hydroxyapatite granules, where needed. In all cases, the implants were placed slightly subcrestal and their primary implant stability was measured by means of RFA; the ISQ values were measured at four sites (buccal/palatal/mesial/distal) in order to calculate the mean ISQ value for each implant. When the mean ISQ < 60, the implants could not be loaded immediately and were therefore considered failed for immediate loading; they were left unloaded placing a transmucosal healing abutment for a period of 3-4 months, during which the patient had to wear a small removable prosthesis, for aesthetic reasons. If the mean ISQ value at placement was ≥60, conversely, the implants were immediately loaded (within 48 hours after implant placement) by means of a single provisional resin crown. A titanium prefabricated abutment was prepared and screwed on the implant; a provisional resin crown was then adapted. The provisional crowns could be obtained from a direct impression (from the laboratory) or from preformed shells which were relined intraorally. Care was taken to polish well all crowns and to obtain a satisfactory, natural emergence profile (Figure 5). In the healed ridge group of patients, interrupted sutures were performed to adapt the flap to the restoration; in the postextraction group, the provisional crown protected the alveolus, maintaining the clot formation subgingivally; in some cases, these crowns could be splinted with composite resin to the adjacent teeth, in the period immediately following the surgery. The provisionals were screw-retained or cemented, depending on the case. A careful check of the occlusion with articulating papers completed the provisional prosthetic phase: light and well distributed static contacts were left, and care was taken to remove any possible overloading. An intraoral periapical radiograph was taken, and the patient was left with prescriptions of oral antibiotics (amoxicillin + clavulanic acid, 2 gr/day for a period of 6 days) and analgesics (600 mg ibuprofen, 2/3 times a day for a maximum period of 2 days). All patients were recalled at 1 week, for a control and the removal of the sutures (where present). The provisional crowns remained in situ (Figure 6) for a period of 3-4 months, after that they were replaced by the definitive ceramic (zirconia ceramic) restorations; the final restorations were cemented (Figure 7). At the delivery of the final crowns, occlusion was carefully checked again, and a new periapical radiograph was taken. All patients were then enrolled in a follow-up program, with visits every 4 months; the patients were followed for a period of 1 year of loading (Figure 8), after the delivery of the final restorations.

### 2.5. Outcomes of the Study

During each follow-up visit (every 4 months) and until the end of the study (1 year after the placement of the definitive crowns) a clinical and radiographic assessment of the implants, peri-implant tissues, and prostheses was carried out by a periodontologist and a prosthodontist, who were not directly involved in the placement of the implants. The main outcomes of the study were implant stability, implant survival, and implant success.

#### 2.5.1. Implant Stability

Resonance frequency analysis (RFA) was the method used to measure implant stability, immediately after placement (primary implant stability) and at each follow-up session. A dedicated instrument (Osstell Mentor®; Osstell, Integration Diagnostic, Sweden) was used to register implant stability. This portable device emitted magnetic pulses to a small magnet (Smartpeg®) screwed directly on the implant with 5 Ncm; the magnet started to vibrate, and the probe listened to the tone and translated it to a value named implant stability quotient (ISQ) [25]. For each fixture, ISQ values (scaled 1–100) were measured from the four sites (mesial, distal, buccal, and palatal sites). The mean of all measurements was rounded to a whole number and regarded as the final ISQ of the implant [25, 26]. At each follow-up session, after each measurement, the abutments were repositioned and screwed again on the implants so that the prostheses could be reinserted. In general, the acceptable stability range is 55–85 ISQ; however, in the present study, in the case of ISQ < 60, implants could not be immediately functionalized/loaded and were therefore considered failed for immediate loading, as previously reported [20].

#### 2.5.2. Implant Survival

A fixture was defined as “surviving” if still present and regular in function, at the end of the study, one year after the placement of the definitive crown. In all cases in which the fixture had to be removed, the implant was defined as “failed.” The causes for which an implant could be removed were (1) lack of osseointegration and mobility, which occurred in the early healing period/provisionalization or even after the placement of the final restoration, but in the absence of symptoms/signs of infection; (2) recurrent and intractable infection of the peri-implant tissues (peri-implantitis) that caused massive bone loss and subsequent implant loosening; (3) fracture of the implant body.

#### 2.5.3. Implant Success

The implant success was defined as the condition in which no biological or prosthetic complications occurred, at the implant and at the restoration level, in the course of the whole study. Among the biological complications, there were (1) postoperative pain/discomfort and edema/swelling; (2) peri-implant mucositis; (3) peri-implantitis; (4) peri-implant bone loss >1.5 mm, without any symptoms or signs of infection, at the 1-year follow-up session. Peri-implant mucositis was defined as a reversible clinical situation in which bleeding on probing and/or suppuration were present, associated with a pocket depth ≥4 mm but with no radiographic bone loss; conversely, peri-implantitis was defined as a nonreversible clinical situation characterized by pocket depth ≥4 mm and bleeding on probing and/or pus secretion associated with evidence of radiographic bone loss (>2.5 mm) [27]. Among the prosthetic complications, there were mechanical complications such as abutment screw loosening and abutment fracture, but also technical complications such as chipping/fracture of the ceramic restorations [2, 28].

### 2.6. Statistical Evaluation

Two independent, experienced observers (a periodontist and a prosthodontist) collected and evaluated all data. Data were entered into a statistical sheet (Excel®, Microsoft, Redmond, USA) where the statistical analysis was performed. The evaluation of patients' demographics (gender, age at surgery, smoking habit, history of periodontal disease, and presence of parafunctions) as well as the implant characteristics (site, position, length and diameter, minor bone augmentation, and connective tissue graft procedures) was carried out. All qualitative variables were evaluated by calculating absolute and relative frequency distributions; the Chi-square test was used to calculate the differences in distribution between the groups, with the significance level set at 0.005. Conversely, quantitative variables (such as patients' age) were analyzed by calculating means, standard deviations (SD), and medians and 95% confidence intervals (CI). Implant survival and success were calculated at the implant level.

## 3. Results

### 3.1. Patient Demographics and Implant Distribution

In total, 34 patients (13 males; 21 females) were enrolled in the present study. The mean age of these patient was 45.58 years (±10.15; median 44; range 20–69; CI 95% 42.14–49.02). The distribution of the patients is reported in Table 1. Although more females were enrolled (21/34: 61.8%), the distribution of patients did not differ significantly in relation to gender (*p* = 0.170). Conversely, most of the selected patients were young adults (with an age comprising between 35 and 49 years, 21/34: 61.8%), with only 7 patients with an age comprising between 50 and 64 years (7/34: 20.6%) and 3 patients with an age < 35 years and ≥65 years (3/34: 8.8%), respectively. Accordingly, the distribution of the patients was nonhomogeneous with respect to the age at surgery (*p* < 0.0001). Most of the patients were nonsmokers (28/34: 82.4%) so that the distribution of patients was not homogeneous with regard to the smoking habit (*p* = 0.0002); however, the percentage of smokers was quite high (6/34: 17.6%). Finally, no statistically significant differences were found in the patients demographics with regard to history of periodontal disease (*p* = 0.303) or presence of parafunctions (bruxism and/or clenching) (*p* = 0.086). In fact, 20 patients had a previous history of chronic periodontal disease (20/34: 58.8%) while 14 patients had not experienced this condition before (14/34: 41.2%). Similarly, 22 patients had no history of parafunctions (22/34: 64.7%), while 12 patients (35.3%) suffered from bruxism and/or clenching.

A total of 43 implants were inserted in this study. Six patients had multiple indications for implant therapy (one patient had four implants installed, another patient received three implants, and four patients had two implants installed). With regard to the distribution of the implants, almost one-third of them were placed in postextraction sockets (14/43: 32.6%), while 29 (29/43: 67.4%) were placed in fully healed sites: these groups did not differ significantly (*p* = 0.022). With regard to the position of the implants, however, a high number of premolars (28/43: 65.1%) were installed, when compared to the incisors (11/43: 25.6%) and with the cuspids (4/43: 9.3%): the distribution of the fixtures in these groups was significantly nonhomogeneous (*p* < 0.0001). No statistically significant differences were found in the distribution of implants by length (*p* = 0.010) and diameter (*p* = 0.026). In almost all cases (37/43: 86.0%) a bone regeneration with autogenous bone particles (collected during the preparation of the implant site) was performed; consequently, there was a significant difference in the distribution of the implants, with regard to the use of bone regeneration procedures (*p* < 0.0001). Finally, in a high number of cases (10/43: 23.3%) a connective tissue graft was harvested from the palate and used to thicken the soft tissues in the buccal area. The connective tissue grafts were placed in almost all cases of central incisors (10/11: 90.9%). In 33 cases, however (33/43: 76.7%) no connective tissue grafts were harvested, and the *p* value observed (0.0005) did not reveal a statistically significant difference in the distribution of the fixtures, with regard to the use of connective tissue grafts. All information about the distribution of the implants is summarized in Table 2.

### 3.2. Implant Stability, Survival, and Success

In the present study, only two implants (2/43: 4.6%) did not show sufficient primary implant (ISQ < 60) and were therefore considered failed for the immediate loading. These implants were not loaded and remained with the healing abutments in position, for a period of 3 months; after this period, they were successfully loaded with a provisional restoration. Both these implants were premolars, placed in the extraction sockets of two different adult patients (49- and 67-year-old females). Conversely, 41 implants (41/43: 95.4%) were satisfactory stable (ISQ ≥ 60) at placement and were therefore loaded immediately.

At the end of the study, one year after the placement of the definitive crowns, no implants failed, for an overall survival rate of 100% (43/43 implants, 41/41 immediately loaded implants in functions).

Finally, with regard to the implant success, no biological complications were reported. In fact, no postoperative pain/discomfort and/or edema/swelling occurred after surgery; in addition, no peri-implant mucositis or peri-implantitis was registered during the entire follow-up period, and the marginal bone loss was <1.5 mm in all implants. However, two prosthetic abutments (2/41: 4.8%) became loose, in two premolars; the abutment screw loosening was registered as prosthetic (mechanical) complication, since it was complication affecting implant components. The abutment screws were tightened again and no other complications occurred at this level. Overall, the rate of complications was therefore 4.8%, for an implant success of 95.2% after 1 year of functional loading.

## 4. Discussion

Nowadays, patients are increasingly demanding and asking for early and immediate prosthetic loading protocols [9, 14]; in the same way, the immediate placement of implants in fresh postextraction sockets represents a valid therapeutic option for the clinician, to reduce the times and costs of implant-prosthetic treatment, the invasiveness of the therapy, and the patient stress [4, 7, 8, 10].

Although the placement of immediate, postextraction implants and the immediate loading protocols can represent today predictable solutions, characterized by high rates of survival and success [7–10], there is no doubt that these methods are more challenging for the clinician, at least when compared to more conventional protocols (such as the insertion of fixtures in fully healed edentulous bone ridges and the conventional, delayed loading after a period of 4–6 months of undisturbed bone healing) [7, 13]. In fact, the placement of implants in extraction sockets can be difficult [7, 13]. First, the postextraction alveolus is generally of larger size than the diameter of the implant: it can therefore be difficult to obtain adequate primary stability of the implant in the surgical site [1, 7, 13]. It is known that primary stability is a fundamental requirement for the survival of the implant, in the short term: an insufficiently stable implant may have a mobilization and failure in the early months of healing, immediately after insertion [17, 18]. In fact, during the first two months following insertion, a bone remodeling with partial loss of initial mechanical stabilization of the implant (resulting from the initial contact between the implant surface and the preexisting alveolar bone) occurs [17, 18]. If this remodeling is not effectively counteracted and balanced by an adequate and rapid deposition of new bone on the implant surface, an adequate secondary stabilization (or osseointegration) of the implant is not possible, with a high risk of failure [17, 18]. Some colleagues have suggested the use of fixtures of larger diameter, in order to get a better primary stability in postextraction sockets: this solution is certainly feasible and viable in the posterior regions [16] but may even be counterproductive in the anterior regions (characterized by high aesthetic impact), where the contact between the implant and the delicate buccal bone plate must be avoided, to prevent the risk of an aesthetic failure [8, 29–31]. For these reasons, generally, the stabilization of the postextraction implants is obtained via an apical preparation that is brought 3-4 mm deeper than the alveolus, for a better apical engagement of the fixture [7, 8, 30, 32, 33]. These surgical strategies are certainly of great validity, but even better results can be obtained if these methods are accompanied by the use of an implant with a design (macrotopography) conceived to maximize the primary stabilization [19, 20, 33].

In the present study on the immediate loading of single implants placed in the anterior areas of the maxilla, almost one-third of all fixtures (32.6%) were placed in postextraction sockets. Despite this, only two implants (4.6%) did not show sufficient primary implant (ISQ < 60) and were therefore considered failed for the immediate loading. This valuable result was certainly possible because long implants were used (72% of the fixtures used in this study were ≥ 13 mm in length) for a better apical engagement and stabilization in the socket; however, the use of tapered implants with knife-edge threads helped to obtain these positive outcomes, because this implant design is potentially able to guarantee a valid primary stabilization even in difficult contexts, as indicated previously in the literature [6, 15, 20].

Immediate loading represents the second possible strategy to reduce the duration of the implant-prosthetic treatment and the cost of therapy: for these reasons, this procedure is more and more appreciated and requested by the patients [9, 16]. Although the immediate loading of implants represents today a reliable procedure in clinically controlled contexts [9], there is no doubt that it could represent a potential risk for treatment failure [17, 18]. In fact, uncontrolled forces and exceeding the physiological limits, transmitted from the crown to the implant, can interfere in the early healing processes at the bone/implant interface and determine the mobilization and failure of the fixture [17, 18].

In our present study, the immediate loading of single implants installed in postextraction sockets and healed sites gave positive clinical outcomes, with high survival rates (100%: no implants were lost during the follow-up). The careful treatment planning and the care and attention devoted to compliance with the strict surgical protocols and prosthetic may explain the excellent results obtained here [34], with a low (4.8%) incidence of complications (no biological complications were reported; only a few prosthetic complications were registered, with two prosthetic abutment loosening encountered during the entire follow-up). However, once again, the use of implants with a specific thread design and macrotopography, able to optimize the primary stabilization, may have contributed to the excellent clinical result obtained in our present work. The threads of the fixtures used in this study, in fact, result in maximized bone-to-implant contact and compressive force resistance and minimized shear force production: this can help in maintaining implant stability in the immediate postplacement healing period, as previously reported in the literature [6, 15, 20]. It should be added, however, that in our present work, all immediately loaded implants were subjected to a controlled load. In fact, all temporary crowns were adjusted with light occlusal marks, so that the occlusal surfaces were in slight static contact with the opposite dentition but with no contact in lateral movements, as previously described in the literature [15]. This procedure allows control and reduces in some way the forces acting on the system, through the prosthetic restoration [15]. In addition, in the present study, some of the single, implant-supported crowns (12/41: 29.2%) were splinted with the neighbouring teeth, for a period of two weeks after surgery. These procedures were particularly important in fresh extraction sockets: in these areas, where the primary stabilization may be more difficult, it is generally considered prudent to avoid overloading the fixture, to prevent the risk of mobilization and failure [17, 18]. In fact, there must be sufficient time to guarantee the transition from the primary (mechanical) stabilization of the implant, to the secondary (biological) stabilization, due to the deposition of new bone directly on the fixture surface [17, 18]. This transition should be as undisturbed as possible, that is, without the occurrence of micromovements at the interface between the bone and the implant, which beyond a certain threshold may interfere with the phenomena of osseointegration [17, 18]. Finally, in the present study, we have used an implant system characterized by a specific micro/nanotopography, with a sandblasted, microstructured surface, which was subsequently treated with incorporation of calcium ions, to become nanostructured. The scientific literature has evidenced that the treatment of implant surfaces stimulates a better and faster bone-implant integration and rapid deposition of new bone on the implant surface [21, 22, 24, 35]. Numerous systematic reviews [21, 22] have shown that the treatment of implant surfaces can be a very important strategy to reduce the conventional healing times, thanks to an increased surface area and surface energy, which are able to promote a better interaction with biological fluids and blood.

The present study has limits, such as the limited number of patients treated (and fixtures inserted) and the short follow-up time. In addition, in this study, only implants placed and immediately loaded in the anterior maxilla have been included. The implants placed in the molar regions of the maxilla were excluded from this evaluation. This is not a trivial matter since in the posterior areas the prosthetic load is higher, and the quality of bone is generally lower: therefore, there may be a higher risk of implant failure, when the immediate functional loading protocol is performed [9, 15, 16]. Finally, it must be pointed out that in the present study in almost all cases of immediate postextraction implants, the provisional resin crowns were splinted with the neighbouring teeth, for a period of two weeks, in order to reduce the possible negative effects of loading (micromotion) at the bone-implant interface, in the first period of healing. In conclusion, the implants inserted in this study should be followed for a longer period of time, and further studies on a larger sample of patients (and possibly including fixtures placed and immediately loaded in the posterior maxilla) will be needed before drawing more specific conclusions.

## 5. Conclusions

In this prospective clinical study with a follow-up of 1 year, the immediate loading of single implants with knife-edge thread design and nanostructured calcium-incorporated surface placed in the anterior maxilla gave positive clinical outcomes, with high survival (100%) and success (95.2%) rates. Only a few, minor prosthetic complications (two abutment screws became loose) were reported, for an overall complication rate of 4.8%. Further long-term studies on a larger sample of patients are needed to confirm these results; in addition, it will be necessary to evaluate the effects of immediate loading on single implants placed in the posterior areas of the maxilla (molar regions), where the prosthetic load is higher.

## Figures and Tables

**Table 1:** Patient demographics.

	Number of patients (%)	*p* ^*∗*^
*Gender*		
Males	13 (38.2%)	0.170
Females	21 (61.8%)	
*Age at surgery*		
20–34 years	3 (8.8%)	<0.0001
35–49 years	21 (61.8%)	
50–64 years	7 (20.6%)	
≥65 years	3 (8.8%)	
*Smoke*		
Yes	6 (17.6%)	0.0002
No	28 (82.4%)	
*History of periodontal disease*		
Yes	20 (58.8%)	0.303
No	14 (41.2%)	
*Parafunctions*		
Yes	12 (35.3%)	0.086
No	22 (64.7%)	

Total	34	—

*p* ^*∗*^ = Chi-square test.

**Table 2:** Distribution of the implants.

	Number of implants (%)	*p* ^*∗*^
*Surgical protocol*		
Postex. sockets	14 (32.6%)	0.022
Healed sites	29 (67.4%)	
*Position*		
Incisors	11 (25.6%)	<0.0001
Cuspids	4 (9.3%)	
Premolars	28 (65.1%)	
*Length*		
10.0 mm	3 (7.0%)	0.010
11.5 mm	9 (20.9%)	
13.0 mm	13 (30.2%)	
15.0 mm	18 (41.9%)	
*Diameter*		
3.5 mm	15 (34.9%)	0.026
4.0 mm	16 (37.2%)	
4.5 mm	8 (18.6%)	
5.0 mm	4 (9.3%)	
*Bone regeneration*		
Yes	37 (86.0%)	<0.0001
No	6 (14.0%)	
*Connective tissue graft*		
Yes	10 (23.3%)	0.0005
No	33 (76.7%)	

Total	43	—

*p* ^*∗*^ = Chi-square test.
